# *Naegleria fowleri* and the future of surveillance: A one-health call to action

**DOI:** 10.1016/j.onehlt.2025.101215

**Published:** 2025-09-19

**Authors:** Anushka Reddy Marri, Davidson H. Hamer, Samendra Sherchan, Kayoko Shioda

**Affiliations:** a Department of Global Health, Boston University School of Public Health, Boston, MA, USA; b Center on Emerging Infectious Diseases, Boston University, Boston, MA, USA; c Section of Infectious Diseases, Department of Medicine, Boston University Chobanian & Avedisian School of Medicine, Boston, MA, USA; d Center for Climate Change and Health, Morgan State University, Baltimore, MD, USA; e Center of Research Excellence in Wastewater-based Epidemiology, Morgan State University, Baltimore, MD, USA

**Keywords:** *Naegleria fowleri*, Primary amoebic meningoencephalitis, Environmental surveillance, One health, Risk mitigation

## Abstract

*Naegleria fowleri* causes primary amoebic meningoencephalitis (PAM), a rapidly fatal central nervous system infection acquired through nasal exposure to contaminated water. PAM cases are rare but increasingly reported across wider geographic areas and through emerging exposure routes in humans and animals. Environmental detections of *N. fowleri* are rising, yet surveillance remains sparse and is often triggered only after clinical cases are identified. This reactive approach delays risk mitigation and limits opportunities for prevention. Rising temperatures, inadequate water treatment, and shifting water use patterns may expand the organism's ecological range. This short communication advocates for proactive environmental and clinical surveillance of *N. fowleri* as a One Health priority. Drawing on global examples, we propose routine monitoring of high-risk water systems, standardization of detection protocols, and case notification requirements. Together with public education in vulnerable settings, these efforts can strengthen preparedness and improve outcomes for a preventable and nearly universally fatal infection.

## Highlights

• *Naegleria fowleri* causes a deadly brain infection with a near 100 % fatality rate.
• The parasite is expanding beyond its historical ecological range.
• Climate change and untreated water increase *N. fowleri* exposure risk.
• We propose proactive testing of high-risk water systems for *N. fowleri*.
• Early detection, reporting, and education can help prevent fatal infections.

## 1 Introduction

*Naegleria fowleri* is a thermophilic, free-living amoeba in the phylum Percolozoa that causes primary amoebic meningoencephalitis (PAM), a rare but near-universally fatal central nervous system infection [1,2]. Transmission occurs through intranasal exposure to *N. fowleri*-contaminated water during recreational activities such as swimming, surfing, or diving, typically affecting immunocompetent children and young adults in summer [1,3]. Symptoms begin with headache, fever, and nausea, rapidly progressing to confusion, seizures, and coma, leading to death within a week [4]. Humans are dead-end hosts with no documented human-to-human spread [4].

*N. fowleri* thrives in warm freshwater and has been detected in both recreational and domestic systems, especially in warm climates and areas with compromised water quality [5]. While predominantly documented in humans, a few documented infections in cattle and wild animals underscore the organism's ecological adaptability and potential for cross-species exposure [6,7].

Although global case numbers remain low, reports show an expanding geographic distribution and emerging exposure routes beyond traditional recreational settings [3,6,8,9]. Recent reviews have advanced understanding of the biology, distribution, and transmission dynamics of *N. fowleri* [6,10]. Building on these insights, this commentary argues for the urgent establishment of proactive, coordinated environmental surveillance, especially in areas facing rising temperatures and vulnerable water infrastructure. Surveillance can enable early detection, risk communication, and timely interventions, potentially averting deaths from a preventable and invariably deadly disease.

## 2 Need for surveillance

Since the first documented case in 1965, around 500 human cases of PAM have been reported globally, with a case fatality rate exceeding 95 % [2,10,11]. Initial infections were reported almost simultaneously in Australia, Florida, and Northern Bohemia in the former Czechoslovakia [[11], [12], [13]]. Since then, most cases have been reported in the United States (USA) and Pakistan, but infections have also occurred across Asia, Mexico, Africa, Europe, and Australasia [6,10]. Many infections are likely undiagnosed due to limited clinical awareness, misdiagnosis as bacterial meningitis, and lack of confirmatory testing, especially in low-resource settings [2]. With under ten documented survivors, most diagnoses occur postmortem [2,11,12]. Animal cases reported in cattle, sheep, a tapir, and a black rhinoceros were also infected through nasal exposure to contaminated water, highlighting that humans and animals alike are exposed through infected environments [6,7].

Multiple environmental and behavioral factors contribute to the organism's emergence and expanding range. Rising surface water temperatures from climate change have been associated with increased *N. fowleri* detection in broader settings, including temperate regions, such as Minnesota (USA), Italy, and Belgium [4,14]. With unchecked greenhouse gas emissions, global models project a surface temperature increase of 3.28 °C by 2100, expanding the ecological niche suitable for *N. fowleri,* which prefers 30–46 °C water [6,15]. Thermal water pollution from industries like power plants, coal, and metallurgical operations has been linked to outbreaks in the Czech Republic and Iraq, creating hotspots where the amoeba may persist year-round [9,15]. Supporting this, the “flagellate-empty habitat” hypothesis suggests that sustained temperature elevation can eliminate microbial competitors, further enabling *N. fowleri* to flourish in these environments [16].

Evolving patterns of human water use have also increased opportunities for exposure. Early cases were linked to recreational exposures such as swimming or bathing in warm lakes or inadequately chlorinated indoor swimming pools [13]. Recent PAM-related pediatric deaths linked to inadequately disinfected splash pads in Arkansas underscore the continued risk from recreational venues [17]. Increasingly, non-recreational exposures, such as nasal irrigation with neti pots, ablution practices, and rinsing sinuses with tap water, have been implicated in documented PAM cases [14,18]. Detection of *N. fowleri* in plumbing, tap water, biofilms, and recreational and non-recreational sources highlights the need to look beyond lakes and rivers [3,6,8].

Although the environmental presence of *N. fowleri* has been increasingly reported, formal, routine surveillance and standard protocols remain extremely limited. In most settings, environmental detection occurs only after a clinical case is identified, reflecting a reactive rather than preventive approach that delays response and limits opportunities for intervention [17]. One notable exception is Louisiana, which has conducted annual *N. fowleri* monitoring in public water systems since 2013 (excluding 2020), as confirmed through correspondence with the Louisiana Department of Health's Safe Drinking Water Program. Each summer, approximately a dozen systems are selected based on factors such as disinfectant residual history, water source type, and use of chloramines or free chlorine. Treated drinking water samples are collected from the distribution system and analyzed using ultrafiltration, culture, microscopy, and qPCR. From 2014 to 2018, *N. fowleri* was detected in nearly 30 % of samples, primarily in sites with low chlorine residual, leading to regulatory changes requiring a minimum disinfectant residual of 0.5 mg/L. In cases of active detection, public notification is issued, and affected systems are instructed to switch to free chlorine and maintain concentrations ≥1.0 mg/L for 60 days before follow-up testing. No active detections have been reported since 2018.

Additional factors such as water stagnation in dead-end lines, low-use zones, aging distribution systems, and the use of chloramines instead of free chlorine may increase contamination risk, underscoring the importance of infrastructure and disinfection choices in surveillance planning [19]. In parallel, case-based surveillance remains fragmented. In the USA, PAM is not a nationally notifiable disease, though it is reportable to state health departments in Florida, Texas, Washington, and Louisiana, which may voluntarily notify the Centers for Disease Control and Prevention (CDC). Confirmed cases are then tracked through the CDC's Free-Living Ameba Surveillance System, though the current status of this system is unclear [2]. To date, no formal environmental surveillance programs or mandatory case reporting systems have been documented elsewhere globally, though recommendations, such as those from the French High Council for Public Hygiene prohibiting swimming when amoebae exceed 100/L of water, have been issued [20].

Compounding these surveillance gaps, limited diagnostic and treatment options add to the challenges. Diagnosis remains challenging due to nonspecific clinical presentations and limited laboratory capacity; the CDC's Free-Living and Intestinal Amebas (FLIA) Laboratory is one of the few facilities in the USA capable of confirming cases and providing urgent diagnostic and therapeutic guidance. While combination regimens including amphotericin B, miltefosine, and azithromycin have shown promise for the treatment of PAM, survival remains rare even with early intervention [21]. Vaccine development efforts, including MP2CL5-based subunit and mRNA platforms, are ongoing but remain in preclinical stages, hindered by immunologic and validation barriers [22]. Widespread vaccination is limited by the low global incidence and geographically dispersed cases, making it difficult to identify a priority population. In the absence of scalable treatment or vaccination strategies, strengthening environmental surveillance and investing in risk communication are the most practical and immediate approaches to reducing missed infections and improving readiness.

## 3 Recommendations

Active clinical and environmental surveillance for *N. fowleri* will require coordination across public health, environmental, and clinical sectors. Priority should be given to high-risk areas with elevated water temperatures, poor chlorination, prior PAM cases, or nasal water exposure practices. Stored or distributed water systems in warm climates may also warrant targeted monitoring. Consistent surveillance will require standardized environmental sampling protocols. These should describe optimal sampling sites, frequency, and key parameters like temperature, turbidity, and free chlorine residual levels. Molecular tools such as qPCR and next-generation sequencing improve detection sensitivity, but use remains limited by cost, infrastructure, and personnel [8,23]. In resource-limited contexts, leveraging basic water quality indicators as initial screening tools can offer a cost-effective option for surveillance.

Clinical surveillance must complement environmental efforts. Due to rapid progression and overlap with other forms of meningitis, PAM is frequently misdiagnosed, especially by providers unfamiliar with the disease. In parallel, designating PAM as a notifiable disease at national or regional levels could improve case detection, enhance data collection, and ensure timely response to future cases.

Finally, incorporating genotyping into surveillance can track geographic strain distribution and detect emerging variants. As recent studies highlight increasing genotypic diversity across regions, including rare strains, these data can inform targeted risk assessments [10]. Public education also plays a critical role. Outreach in high-risk settings, focusing on practices such as ablution, neti pot use, and recreational freshwater activities, together with education about low-cost methods such as safe water (distilled, boiled, or filtered) for nasal irrigation and avoiding submersion or using nose clips while swimming, can reduce household- and community-level risk when paired with broader surveillance [18,19].

## 4 Conclusion

The outlook for people who contract PAM is bleak, given the lack of effective treatment, and the infection risk is global. Continued detection of *N. fowleri* in diverse water sources, coupled with the lack of routine surveillance, highlights a critical gap in public health preparedness. As rising surface temperatures, inadequate water treatment, and increasing human activities in contaminated water expand the pathogen's reach, integrating *N. fowleri* into environmental and clinical monitoring systems becomes essential. Although infections remain rare, their near-universal fatality demands proactive action. Classifying PAM as a notifiable disease and embedding *N. fowleri* surveillance into existing systems are immediate, feasible steps. These efforts, paired with provider-focused health education in high-risk areas, can save lives by improving diagnosis and strengthening resilience at the intersection of environmental, animal, and human health.

## CRediT authorship contribution statement

**Anushka Reddy Marri:** Writing – review & editing, Writing – original draft, Conceptualization. **Davidson H. Hamer:** Writing – review & editing, Supervision, Conceptualization. **Samendra Sherchan:** Writing – review & editing. **Kayoko Shioda:** Writing – review & editing, Supervision, Conceptualization.

## Funding

This research did not receive any specific grant from funding agencies in the public, commercial, or not-for-profit sectors. This commentary is supported by the Center on Emerging Infectious Diseases (CEID) at Boston University.

## Declaration of competing interest

The authors declare that they have no known competing financial interests or personal relationships that could have appeared to influence the work reported in this paper.

## Data Availability

No data was used for the research described in the article.
